# Cost effectiveness analysis of immunotherapy regimens currently approved in advanced or recurrent endometrial cancer: An analysis of the NRG-GY 018, RUBY, and DUO-E trials

**DOI:** 10.1016/j.gore.2026.102050

**Published:** 2026-02-18

**Authors:** Alex A. Francoeur, Su-Ying Liang, Brittany File, Eunji Choi, Max Brameld, Michael T. Richardson, Caitlin R. Johnson, Daniel S Kapp, Krishnansu S. Tewari, John K. Chan

**Affiliations:** a Department of Obstetrics and Gynecology, University of California, Irvine, Orange, CA, USA; b Palo Alto Medical Foundation Research Institute, Sutter Health, Palo Alto, CA, USA; c Department of Population Health Sciences, Weill Cornell Medicine, New York, NY, USA; d Department of Obstetrics and Gynecology, University of California Los Angeles, Los Angeles, CA, USA; e Department of Radiation Oncology, Stanford University School of Medicine, Stanford, CA, USA; f Division of Gynecologic Oncology, California Pacific / Palo Alto / Sutter Health Research Institute, San Francisco, CA, USA

**Keywords:** Cost effectiveness, Immunotherapy, Pembrolizumab, Dostarlimab, Durvalumab, Advanced endometrial cancer, Recurrent endometrial cancer

## Abstract

**Background:**

The purpose of this project was to evaluate the cost-effectiveness of novel FDA approved regimens incorporating immunotherapy into upfront treatment with chemotherapy in advanced or recurrent endometrial cancer in the mismatch repair deficient (dMMR) and mismatch repair proficient (pMMR) cohorts.

**Methods:**

Partitioned survival models were established based on the RUBY, NRG-GY 018, and DUO-E trials, as well as available FDA data. Incremental cost-effectiveness ratios (ICERs) were estimated based on quality adjusted progression free life years saved (QA-PFLYS). Tree Age software was used to perform partitioned survival modeling. Outcomes for dMMR and pMMR groups were compared based on the current FDA approvals. Costs were obtained from Redbook Micromedex pricing and adjusted for inflation from study activation to public dissemination of trial results.

**Results:**

The addition of pembrolizumab, dostarlimab, or durvalumab to chemotherapy in patients with dMMR tumors resulted in improvements in progression free life years saved. Based on QA-PFLYS, the ICER for pembrolizumab was $217,713, followed by $351,280 for durvalumab, and $451,750 for dostarlimab. When continuing durvalumab for 2 years, the ICER was $262,087. When looking at patients with pMMR tumors, the ICER for pembrolizumab was $250,535 while the ICER for dostarlimab was $1,349,776.

**Conclusions:**

Recognizing existing differences in study design, both pembrolizumab and durvalumab may be more cost effective than dostarlimab in our model. This appears to largely be driven by differences in duration of maintenance therapy rather than differences in effectiveness or individual drug cost. Immunotherapy does not appear to be as cost effective in the pMMR population.

## Highlights

• Immunotherapy has become standard of care in advanced and recurrent endometrial cancer with multiple FDA approvals.
• A cost effectiveness analysis was done with a partitioned survival model in FDA approved regimens for endometrial cancer.
• Pembrolizumab appears to be more cost effective than dostarlimab and durvalumab but all regimens are costly.
• More work is needed to improve the cost of novel therapeutics.

## 1 Introduction

Uterine cancer is the most common female pelvic cancer in the United States with approximately 66,000 cases expected to be diagnosed in 2025 (Siegel et al., 2024). It is usually diagnosed at an early stage and patients can often be cured with surgery, with 5-year survival rates of 95% in patients with localized disease. Treatment for advanced uterine cancer typically requires a combination of chemotherapy, radiation, and/or surgery and has a much lower 5-year survival rate of 20% (Cancer Stat Facts: Uterine Cancer, 2024).The standard of care therapy in the front line and recurrent setting has been doublet chemotherapy carboplatin and paclitaxel which has a demonstrated overall survival (OS) of 37 months based on GOG-209 (Miller et al., 2020). Of note, this trial was conducted from 2003 to 2009, included a larger proportion of stage 3 disease, and did not allow for prior chemotherapy in contrast to the below trials.

Since 2023, there have been multiple prospective randomized trials published or presented on the adoption of immune checkpoint inhibitors (ICI) with chemotherapy in advanced or recurrent endometrial cancer with subsequent FDA approval of 3 medications (Mirza et al., 2023, Eskander et al., 2023, Westin et al., 2024). In July 2023, the FDA approved dostarlimab in combination with carboplatin and paclitaxel, followed by dostarlimab maintenance, for primary advanced or recurrent endometrial cancer that is mismatch repair deficient (dMMR). Subsequently, in August of 2024, dostarlimab use was FDA approved in an all-comer population. The RUBY trial investigators (NCT #03981796) demonstrated that patients with dMMR/microsatellite instability-high (MSI-H) tumors who were treated with dostarlimab had a 71% reduction (Hazard ratio (HR) = 0.29, 95% CI: 0.16–0.50) in the risk of progression or death (Mirza et al., 2023). Pembrolizumab in combination with chemotherapy was approved by the FDA for advanced and recurrent endometrial cancer in June 2024 for both dMMR and pMMR tumors based on the results of the NRG GY018 trial (NCT #03914612). Investigators found a 70% reduction (HR = 0.30, 95% CI: 0.19–0.48) in the risk of progression compared to chemotherapy in the dMMR cohort. In the mismatch repair proficient (pMMR) cohort, pembrolizumab led to a 46% risk reduction (HR = 0.54, 95% CI: 0.41–0.71) compared to chemotherapy alone (Eskander et al., 2023). In June 2024, durvalumab in combination with chemotherapy was also approved for dMMR advanced and recurrent endometrial cancer based on the DUO-E trial (NCT #04269200) reporting a 58% reduction in the risk of progression or death (HR = 0.42, 95% CI: 0.22–0.80) (Westin et al., 2024).

Cancer directed therapeutics are a major source of healthcare cost in the United States and are projected to increase by over $63 billion dollars by 2030 (Mariotto et al., 2020). This cost comes with increasing financial burden to cancer patients and their families. A study of 9.5 million patients with newly diagnosed cancer found that 42.4% depleted their life’s savings after only 2 years of treatment (Gilligan et al., 2018). Cost is multifactorial and can include outpatient co-pay, inpatient admission cost, imaging cost, as well as cost of anti-cancer therapeutics (Aviki et al., 2022). Prior cost-effectiveness studies examining immunotherapy in advanced and recurrent endometrial cancer have not compared all three of the above regimens (Coleman et al., 2025, Huo et al., 2024, You et al., 2023, Kim et al., 2023). The objective of the present study was to examine the cost-benefit of incorporating immunotherapy into the treatment of advanced and recurrent endometrial cancer based on current FDA indications for use.

## 2 Methods

### 2.1 Model

**Fig. 1:**
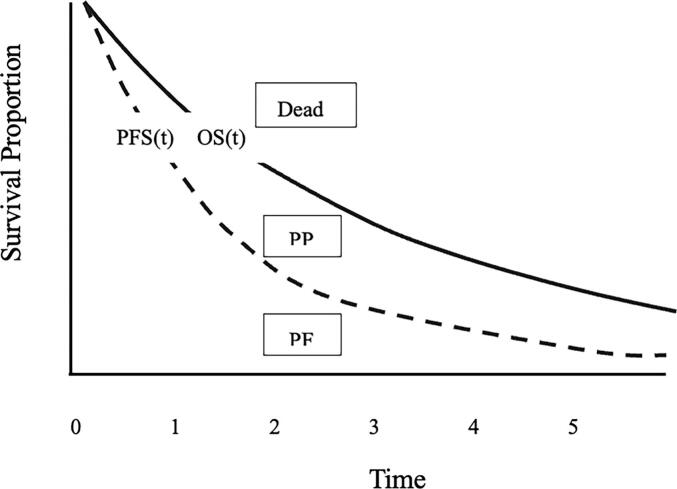
Example of a partitioned survival model, using overall survival (OS) and progress-free survival (PFS) curves to determine a three-state membership at a given time. Abbreviations PF: progression-free; PP: post-progression. PP(t)=OS(t)-PFS(t).

A partitioned survival decision model was created to simulate the clinical trajectory of a hypothetical cohort of women with advanced or recurrent endometrial cancer (Fig. 1). The partitioned survival analysis approach uses two survival curves, overall survival (OS) and progression-free survival (PFS), to partition three mutually exclusive health states (progression-free, post-progression, dead) at each time point. PFS curves directly provide the proportion of patients remaining in the healthy, progression-free state. The proportion of patients in the post-progression state is derived as the difference between the OS and PFS, while proportion of patients in the dead state is calculated as 1 minus the OS curve at each time point (Rui et al., 2021). Given limited long term survival follow up and wide ranges of trial crossover, the partitioned survival model was determined to better estimate cost effectiveness with the known data available (Rui et al., 2021, Woods et al., 2020). We compared treatment with dostarlimab in combination with carboplatin and paclitaxel (DOS-TC), pembrolizumab in combination with carboplatin and paclitaxel (PEM-TC), durvalumab in combination with carboplatin and paclitaxel (DUO-TC), or carboplatin and paclitaxel alone (TC). Cohort size was not needed as the portioned survival model uses proportions of patients in each disease state to compute costs (Siegel et al., 2024, Rui et al., 2021). We estimated a dMMR rate of 33% and a pMMR rate of 67% based on available data (Fung-Kee-Fung et al., 2006, Kandoth et al., 2013). Given differences seen in the dMMR and pMMR populations, cost effectiveness analyses were separated based on mismatch repair status.

Cycle length was determined to be 1-year increments based on the available published data (Mirza et al., 2023, Eskander et al., 2023, Westin et al., 2024). The time horizon was set at 5 years as historically, over 70% of advanced or recurrent endometrial cancer will relapse by that time (Miller et al., 2020). In the model, pembrolizumab was administered with carboplatin and paclitaxel for 6 cycles followed by maintenance pembrolizumab for a maximum total of 2 years based on the NRG-GY018 published data. Dosing of pembrolizumab was 200 mg every 3 weeks for 6 cycles, subsequently it was 400 mg every 6 weeks for up to 2 years (Eskander et al., 2023). Dostarlimab was administered at a dose of 500 mg every 3 weeks with carboplatin and paclitaxel for 6 cycles, subsequently, it was given at 1000 mg every 6 weeks for up to 3 years (Mirza et al., 2023). Durvalumab was given at 1;120  mg every 3 weeks with carboplatin and paclitaxel for 6 cycles and subsequently at a dose of 1,500 mg every 4 weeks continued until progression or toxicity (Westin et al., 2024). A 2-year and 3-year parameter was set for durvalumab to allow comparison to pembrolizumab and dostarlimab dosing. Dosing of carboplatin was area under the curve 5, and paclitaxel 175 mg/m^2^ body surface area every 3 weeks for 6 cycles. Patients who responded to treatment were continued until progression or death. The model treated patients with 3 cycles of the assigned therapy prior to any progression based on standard practices.

Effectiveness was measured in quality adjusted progression free survival life years saved (QA-PFLYS). QA-PFLYS was chosen given significant trial crossover in some of the trials potentially confounding OS results. Prior studies with limited or confounded OS data have chosen this approach (Smith et al., 2015). Quality adjusted life year (QALY) saved data is reported in a supplement. Incremental cost-effectiveness ratios (ICERs) were expressed in 2024 US dollars per QA-PFLYS gained. The ICER was defined as the difference in total cost divided by the difference in QA-PFLYS for each of the strategies. We assumed a willingness-to-pay threshold (WTP) of $100,000/QA-PFLYS and considered an intervention to be cost-effective if its ICER was below this value. Models were created and analyzed using TreeAge Pro 2024, TreeAge Software, Williamstown, MA.

### 2.2 Clinical estimates

The inputs for the model were based on the published literature (Table 1). The toxicity rate was estimated by the rates of grade 3 or higher toxicities for patients with dMMR and pMMR endometrial cancer in each trial. If the trials were not grouped by MMR status, it was assumed the toxicity rate was as reported for the whole group. Survival outcomes were estimated from the PFS published data by digitizing the progression free survival Kaplan Mayer curves for each respective study population.

**Table 1:** Cost, toxicity, and utility parameters for the partitioned survival model.

Treatment (per cycle)	Probability	Cost USD (2024 per cycle)	Utility	Reference
Dostarlimab + carboplatin + paclitaxel		14,462		(Mirza et al., 2023)
Pembrolizumab + carboplatin + paclitaxel		13,464		(Eskander et al., 2023)
Durvalumab + carboplatin + paclitaxel		10,146		(Westin et al., 2024)
Carboplatin + paclitaxel		1,126		(Mirza et al., 2023, Eskander et al., 2023, Westin et al., 2024)
**Grade 3 or higher toxicity**				
Dostarlimab + carboplatin + paclitaxel	0.71	9,784	0.70	(Mirza et al., 2023, Dioun et al., 2023, Wu et al., 2018)
Pembrolizumab + carboplatin + paclitaxel	0.63	9,784	0.70	(Eskander et al., 2023, Dioun et al., 2023, Wu et al., 2018)
Durvalumab + carboplatin + paclitaxel	0.54	9,784	0.70	(Westin et al., 2024, Dioun et al., 2023, Wu et al., 2018)
Carboplatin + paclitaxel	0.53	9,163	0.70	(Mirza et al., 2023, Eskander et al., 2023, Westin et al., 2024, Dioun et al., 2023, Wu et al., 2018)
**Cancer cost and utility**				
Receiving chemotherapy			0.84	(Taylor et al., 2019, Yabroff et al., 2008)
Progressive disease			0.50	(Taylor et al., 2019, Yabroff et al., 2008)

### 2.3 Costs

Costs of drugs were obtained using Micromedex RedBook and reported in inflation adjusted 2024 US dollars (Red, 2025). The estimated cost of carboplatin, paclitaxel and dostarlimab is $14,462/cycle (q3weeks, 6 total cycles) and that of carboplatin, paclitaxel and pembrolizumab is $13,464; the estimated cost of carboplatin and paclitaxel alone is $1,126 per cycle (q3weeks, 6 total cycles). The estimated cost of carboplatin, paclitaxel and durvalumab is $10,146/cycle (q3weeks, 6 total cycles). The estimated cost of maintenance dostarlimab is $26,672/cycle (q6weeks for up to 3 years or up to 42 additional cycles), the estimated cost of maintenance pembrolizumab is $24,676 per cycle (q6weeks for up to 2 years), and the estimated cost of maintenance durvalumab is $12,081/cycle (q4weeks until progression or toxicity).

Cost for complications and grade 3 or higher adverse events were estimated using the existing published study data and previously published studies examining dostarlimab, pembrolizumab, and first- and second-line chemotherapy (Mirza et al., 2023, Eskander et al., 2023, Westin et al., 2024, Dioun et al., 2023, Wu et al., 2018).

### 2.4 Quality of life

Quality of life was accounted for by adjusting the utility ratio. Patients receiving active treatment were given a utility ratio of 0.80. Progressive disease had a utility ratio of 0.50. Parameters are reported in Table 1.

### 2.5 Sensitivity Analysis

A one-way sensitivity analysis was performed to determine the cost at which each drug regimen would be considered cost effective using a willingness to pay threshold of $100,000 based on prior similar studies (Dioun et al., 2023, Wu et al., 2018). Results were stratified by MMR status. A univariate sensitivity analysis was performed for dMMR DOS-TC vs TC and DUO-TC vs TC (2 and 3 year) along with pMMR PEM-TC vs TC and DOS-TC vs TC on key model inputs that included the costs of regimen, response rates, costs for toxicities, and health utility values, which are displayed in a tornado diagram. This was done by modifying one of the above parameters at a time to determine the effect on the ICER. Probabilistic sensitivity analysis were performed by Monte Carlo simulation by determining the probability of each treatment to be cost effective at different payer thresholds calculated over 1,000 simulations. This data was shown as a cost effectiveness acceptability curve and probability distribution. A CHEERS checklist was completed for the above study (Husereau et al., 2022).

## 3 Results

### 3.1 DMMR patients

Using a partitioned progression free survival 5-year time horizon model, for patients with dMMR tumors, the use of immunotherapy provided improvements in progression free quality adjusted life years. The addition of pembrolizumab to standard chemotherapy resulted in an incremental effectiveness of 1.385 years. The use of dostarlimab had an incremental effectiveness of 1.053 years. Finally, the addition of durvalumab to standard chemotherapy had an incremental effectiveness of 0.907 years. When looking at cost, chemotherapy remains the least costly treatment option in these patients with a total cost of $19,259. Pembrolizumab adds an incremental cost of $301,437, dostarlimab adds an incremental cost of $475,703, and durvalumab adds an incremental cost of $237,792 for two years of maintenance and $318,717 for three years of maintenance. Based on QA-PFLYS, the ICER for pembrolizumab was $217,713, followed by $351,280 for durvalumab, and $451,750 for dostarlimab. When continuing durvalumab for 2 years, the ICER was $262,087 compared to standard chemotherapy. If dostarlimab was continued for 2 years instead of 3, the ICER would be $314, 983.

### 3.2 PMMR patients

When looking at patients with pMMR tumors, pembrolizumab resulted in an incremental effectiveness of 0.960 years. Pembrolizumab added an incremental cost of $240,414 to the treatment of these patients. The QA-PFLYS ICER for pembrolizumab was $250,535. The incremental effectiveness of dostarlimab for pMMR tumors was 0.283 years. There was a total incremental cost of $381,797 with an ICER of $1,349,776 in this patient population. If dostarlimab was continued for 2 years maintenance instead of 3, the ICER would be $1,039,398. The results of the cost effectiveness analysis are summarized in Table 2.

**Table 2:** Incremental cost effectiveness reported in quality adjusted progression free life years saved (QA-PFLYS) per each FDA approved regimen for advanced or recurrent endometrial cancer by mismatch repair status.

				dMMR subgroup					pMMR subgroup			
Regimen	PEM-TC	DOS-TC (2-year)	DOS-TC (3- year)		DUO-TC (2-year)	DUO-TC (3-year)	TC	PEM-TC		DOS-TC (2-year)	DOS-TC (3-year)	TC
Total Cost ($)	320,696	354,169	498,188		256,213	337,138	19,259	259,662		316,647	404,440	19,248
Incremental Cost ($)	301,437	331,684	475,703		237,792	318,717		240,414		294,004	381,717	
Incremental effectiveness	1.385	1.053	1.053		0.907	0.907		0.96		0.283	0.283	
PFLY	3.303	3.928	3.928		3.023	3.023		2.371		2.693	2.693	
ICER ($/QA-PFLYS)	217,713	314,983	451,750			351,280		250,535		1,039,398	1,349,776	

*Abbreviations*: TC, carboplatin-paclitaxel; PEM − pembrolizumab; DOS-dostarlimab; DUO-duvarlumab; dMMR-mismatch repair deficient; pMMR-mismatch repair proficient; PFS-LYS, progression-free life years saved; QALY, quality-adjusted life years; ICER, incremental cost-effectiveness ratios.

## 4 Sensitivity analysis

**Fig. 2:**
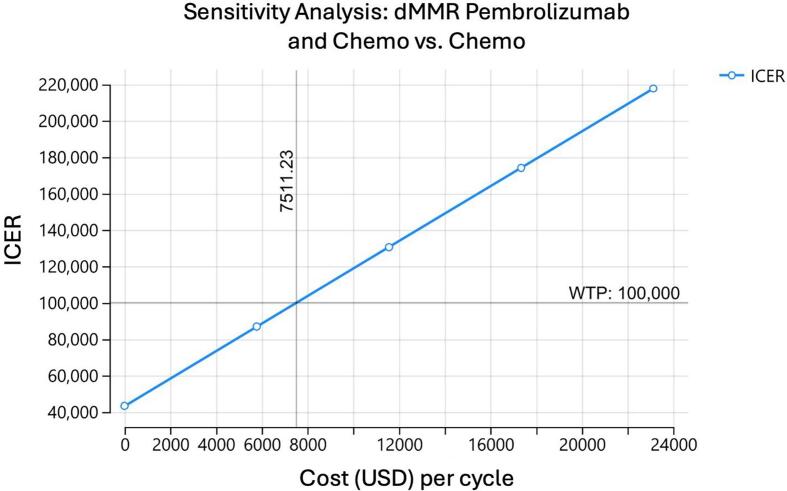

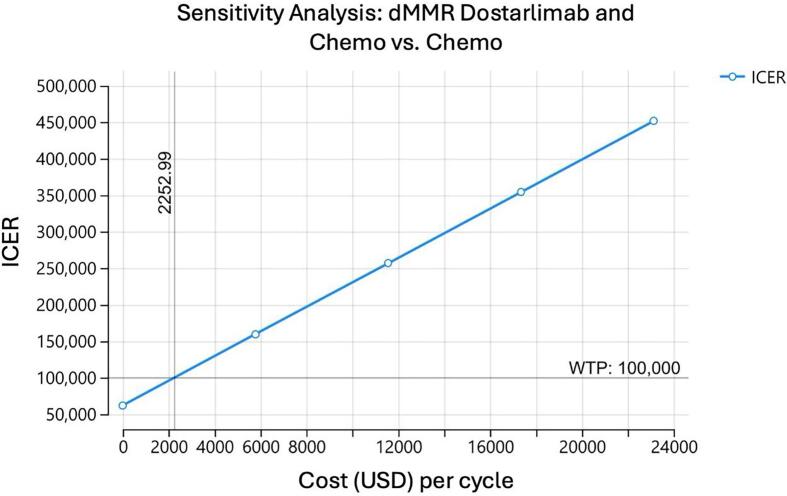

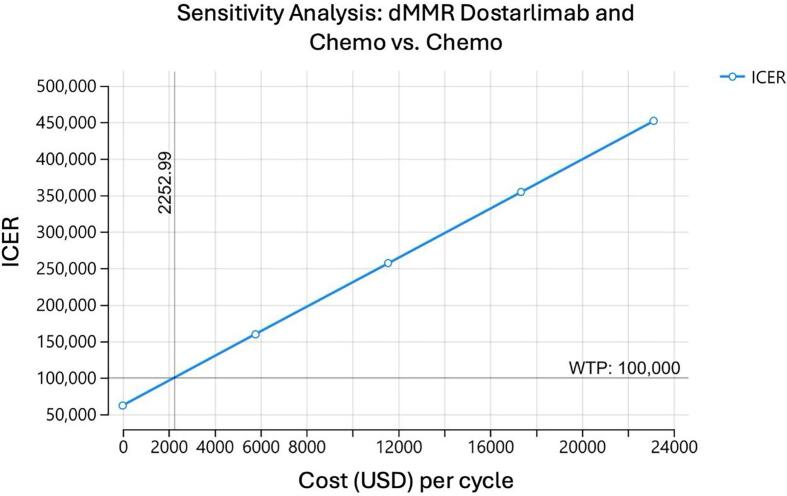

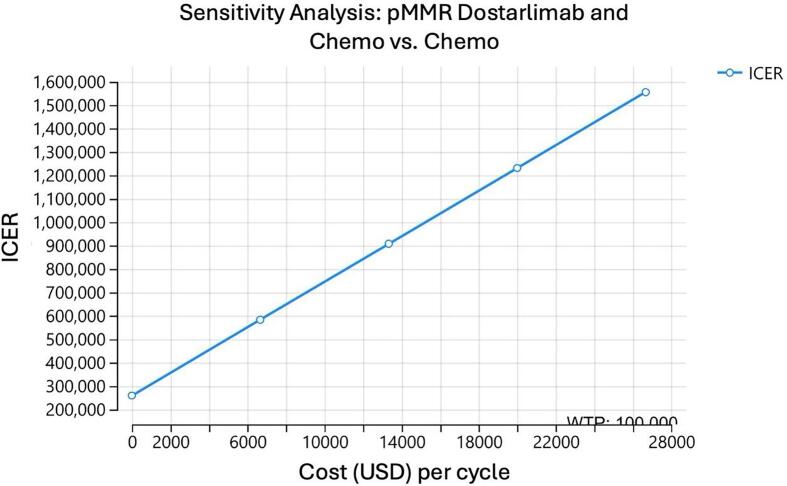

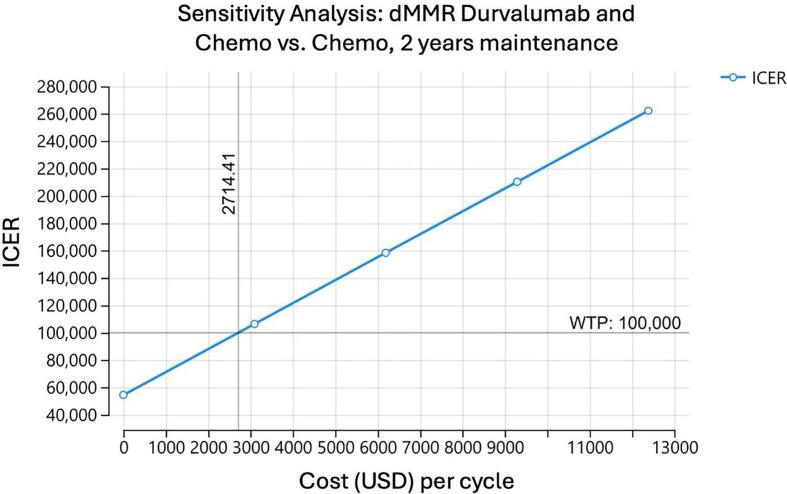

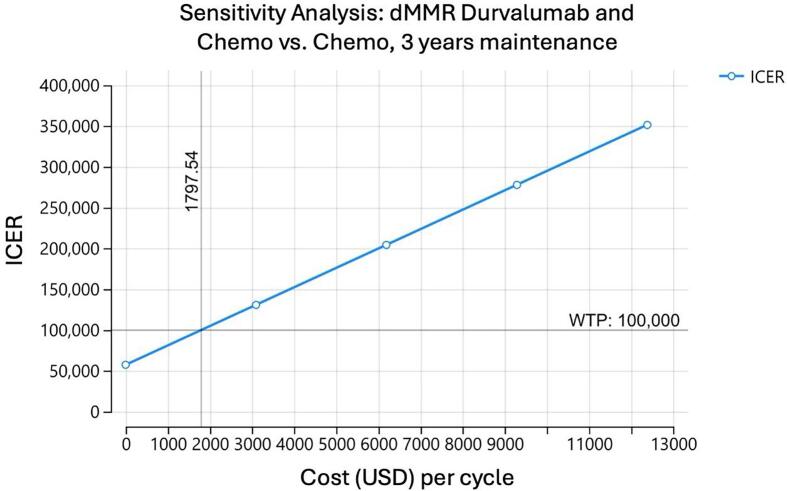
One-way sensitivity analysis of each model assessing at what cost each medication regimen could be considered cost effective assuming a willingness to pay threshold of $100,000. A. Sensitivity analysis of pembrolizumab and chemotherapy for patients with dMMR mutations. B. Sensitivity analysis of pembrolizumab and chemotherapy for patients with pMMR mutations. C. Sensitivity analysis of dostarlimab and chemotherapy for patients with dMMR mutations. D. Sensitivity analysis of dostarlimab and chemotherapy for patients with pMMR mutations. E. Sensitivity analysis of durvalumab and chemotherapy for patients with dMMR mutations with 2 years of maintenance. F. Sensitivity analysis of durvalumab and chemotherapy for patients with dMMR mutations with 3 years of maintenance.

A one-way sensitivity analysis was performed on the model to determine at what cost each medication regimen could be considered cost effective. For pembrolizumab to be cost effective for a dMMR population, assuming a willingness to pay (WTP) of 100,000 dollars, the cost of the drug would have to be $7,511 dollars. In other words, the cost of the medication would have to decrease by 4,827 dollars per cycle (Fig. 2A). In comparison, for pembrolizumab to be effective in the pMMR population, the cost of the drug would have to be 5,171 dollars per cycle, or a decrease in cost of 7,167 dollars per cycle (Fig. 2B). When examining dostarlimab, the cost of the drug would have to decrease to 2,253 dollars per cycle to be cost effective for the dMMR population, a decrease of 11,083 dollars per cycle (Fig. 2C). We were unable to calculate an effective cost of dostarlimab for the pMMR population (Fig. 2D). For durvalumab, an assumption was made of maintenance for 2 or 3 years for the dMMR population. For two years of maintenance, the cost of durvalumab would have to decrease to 2,714 dollars per cycle, or a decrease of 6,306 dollars per cycle (Fig. 2E). If durvalumab was continued for 3 years as maintenance, the cost would have to be decreased to 1,798 per cycle, a decrease of 7,222 dollars per cycle (Fig. 2F).

A Monte Carlo sensitivity analysis was performed to predict the reliability of the model (Supplemental Fig. 1A-F). A tornado chart for each regimen is included in the Appendix (Supplemental Fig. 2A-F). The cost and duration of immunotherapy was the major driver in the ICER for each drug.

## 5 Discussion

### 5.1 Summary of results

In this cost-effectiveness analysis examining novel treatments for advanced and recurrent endometrial cancer, we found standard chemotherapy to be more cost-effective when compared to the addition of immunotherapy largely driven by the cost of the addition of immunotherapy. Pembrolizumab, dostarlimab, and durvalumab all significantly improve PFS in patients with dMMR tumors but at a higher cost. Pembrolizumab would be more cost-effective than dostarlimab or durvalumab if the cost of the medication decreased or if the willingness to pay threshold increased. The incremental cost of dostarlimab compared to pembrolizumab appears to be driven by the additional year of maintenance as demonstrated by lower cost with 2 years of modeled maintenance.

### 5.2 Results in context

Novel and targeted therapies have been shown to improve progression free and overall survival in patients with gynecologic malignancies but typically come at a high cost compared to standard treatment. For example, previous studies examining the addition of targeted agents to treatment of cervical and ovarian cancer found ICERs in the range of $119,000-$320,000 per QALY. In endometrial cancer specifically, previous studies have looked at the cost effectiveness of second line treatment with dostarlimab, pembrolizumab, or pembrolizumab-lenvatinib. Dostarlimab in the second line was found to have an ICER of $331,913 per QALY compared to chemotherapy (Dioun et al., 2023). In a study of pembrolizumab in the second line, dMMR patients had an ICER of $147,249 compared to pMMR patients with an ICER of $341,830 per QALY (Barrington et al., 2019).

A recent paper by Coleman et al. examined the cost-effectiveness of dostarlimab plus chemotherapy for patients with primary advanced and recurrent endometrial cancer in both dMMR and all-comer populations, based on the current indication for the drug (Coleman et al., 2025). They similarly performed a partitioned survival model and reported an ICER of $57,151/QALY for dostarlimab in the dMMR population and $143,783/QALY for dostarlimab in the overall population. The model used a lifetime time horizon which may account for the differences seen. Given the short long term follow up, it is hard to predict lifetime cost-effectiveness for these treatments so, while our model may overestimate cost, their model may underestimate it. Further, the overall population includes both patients with dMMR and pMMR tumors and this may confound the ICER ratios found when predicting cost effectiveness for patient with pMMR tumors.

An additional three studies utilized Markov modeling in their cost-effective analyses for immunotherapy in advanced and recurrent endometrial cancer (Huo et al., 2024, You et al., 2023, Kim et al., 2023). Markov modeling requires additional assumptions to be made regarding health states whereas the partitioned survival model uses individual data to construct the model, making partitioned survival modeling helpful in cases where limited long term follow up exists (Rui et al., 2021). Kim et al. examined the cost effectiveness of dostarlimab and pembrolizumab in addition to chemotherapy in the upfront setting based on the NRG-GY018 and RUBY trials (Kim et al., 2023). In the dMMR subpopulation, in both study arms these authors reported an ICER of $377,718/QALY for pembrolizumab and an ICER of $401,859/QALY for dostarlimab. However, researchers did not look at the use of durvalumab in this patient population. Despite the use of Markov modeling, our study found similar results to Kim et al. when using the partitioned survival model. Additionally, Kim et al. focused their analysis on the dMMR patient population, while we examined differences in both the dMMR and pMMR groups. Huo et al. conducted a cost-effectiveness analysis of dostarlimab plus chemotherapy in the same group, but stratified by dMMR and pMMR subgroups (Huo et al., 2024). They reported an ICER of $60,349.30/QALY in the dMMR group and $175,788.47/QALY in the pMMR group. This discrepancy in findings could be due to use of the Markov modeling or in their attempt to extend the survival curve discrepancy noted when comparing the reconstructed survival curve with the actual data.

Lastly, You et al. examined cost effectiveness of dostarlimab in the dMMR, pMMR, and overall populations in the same upfront setting (You et al., 2023). The reported ICER of dostarlimab plus chemotherapy was $53,063.61/QALY for the dMMR subgroup, $124,088.56/QALY for the pMMR subgroup, and $98,276.61/QALY for the overall population. This analysis is difficult to compare accurately given their analysis is performed from the perspective of the Chinese healthcare system utilizing different treatment related costs and WTP thresholds.

Rising costs in cancer care are widespread and a large proportion of cost is attributable to therapeutics (Zaorsky et al., 2021, Hong et al., 2018, Mailankody and Prasad, 2015). Discussions of cost are invariably connected to the willingness to pay threshold. Historically the WTP threshold was considered to be $50,000/QALY based off of the cost ratio of renal dialysis, with more updated research bringing that level to $100,000–150,000/QALY (Grosse, 2008, Hirth et al., 2000, Winkelmayer et al., 2002). Patients treated with immunotherapy can have variable but, in some cases, durable responses that are more challenging to quantify with ICER ratios (Green, 2021). It is unclear what the WTP threshold should be for novel cancer therapeutics such as immunotherapies. One study suggested consideration of a WTP of $300,000/QALY for anti-cancer medications (Nadler et al., 2006).

One major difference between the RUBY, GY-018, and DUO-E trials was the duration of maintenance therapy. The RUBY trial continued dostarlimab maintenance for 3 years, while pembrolizumab is continued for 2 years in the GY-018 trial, and durvalumab was continued until progression or unacceptable toxicity in DUO-E. The differences in maintenance duration may contribute to the differences in ICER seen. The optimal duration for maintenance in immune checkpoint inhibitors is not yet established. Research recommends a duration of anywhere from 1 year to continuation until progression depending on the disease process being studied (Yin et al., 2022). Studies are investigating the optimal duration of immunotherapy with the Safe Stop trial investigating discontinuation of immunotherapy after 1 year if complete or partial response in patients with melanoma (Mulder et al., 2021). Additionally, high costs of these drugs can prevent further research as cost of conducting a comparative effectiveness study could be prohibitive (Mailankody and Prasad, 2014).

This study examined the cost effectiveness of dMMR and pMMR patients in relation to standard chemotherapy but did not examine differences in cost between other immunotherapy-based regimens for pMMR patients such as pembrolizumab and lenvatinib as in KEYNOTE-775 (Makker et al., 2022). If pMMR patients are treated with upfront chemotherapy and progress, they could then be treated with pembrolizumab and lenvatinib if they subsequently develop recurrent disease. Thus, a more detailed cost effectiveness analysis looking at the effectiveness of upfront immunotherapy in this population is warranted.

### 5.3 Strengths and limitations

This study has several strengths and limitations. The results of this study are based on published results from the RUBY, GY-018, and DUO-E trials. The partitioned survival model requires certain assumptions including assumptions related to health utility states and toxicity, which may not always accurately reflect real world outcomes. Our study ran our model for a 5-year time but follow up data is not fully mature yet. This model chose to use QA-PFLYS instead of quality of life adjusted survival due to differences in study crossover at progression which could limit long term interpretation of results. Each trial had different inclusion and exclusion criteria which could limit cross trial comparison of cost effectiveness. The NCCN lists the combination of chemotherapy, durvalumab and olaparib as an option for the pMMR subgroup. Analysis of the cost-effectiveness of this combination could be performed in future work. However, this model is strengthened by its use of the partitioned survival model which, in the case of limited long term follow up, requires fewer assumptions compared to the Markov model. Long term overall survival data may impact these models as the data have time to mature, however may be limited by trial arm crossover. Additionally, this is only the second study to examine the cost effectiveness of these novel regimens in the pMMR population and the first study to examine all FDA approved regimens containing immunotherapy for advanced and recurrent endometrial cancer, best reflecting real world uses of these medications.

## 6 Conclusions

This model examining cost effectiveness in recurrent and advanced endometrial cancer finds that pembrolizumab is more cost effective than durvalumab and dostarlimab in both the populations they are approved for, however all exceed the traditional willingness to pay threshold. Regardless, the benefit of immunotherapy in this population is pronounced, especially in the dMMR population and merits its use. Patients with pMMR tumors seem to derive less benefit and incur more cost from use of immunotherapy. This study demonstrates the need to establish an optimal duration of maintenance therapy with novel therapeutics and approaches to introduce cost mitigation strategies to cancer care delivery.

## CRediT authorship contribution statement

**Alex A. Francoeur:** Writing – original draft, Visualization, Conceptualization. **Su-Ying Liang:** Software, Methodology, Formal analysis. **Brittany File:** Writing – original draft. **Eunji Choi:** Software, Methodology, Formal analysis. **Max Brameld:** Software, Methodology, Formal analysis. **Michael T. Richardson:** Writing – review & editing. **Caitlin R. Johnson:** Writing – review & editing, Project administration. **Daniel S Kapp:** Writing – review & editing. **Krishnansu S. Tewari:** Writing – review & editing. **John K. Chan:** Writing – review & editing, Supervision, Funding acquisition, Conceptualization.

## Declaration of competing interest

The authors declare that they have no known competing financial interests or personal relationships that could have appeared to influence the work reported in this paper.
