# Supplementary material for: Cost effectiveness analysis of immunotherapy regimens currently approved in advanced or recurrent endometrial cancer: An analysis of the NRG-GY 018, RUBY, and DUO-E trials
Source: Gynecol Oncol Rep. 2026 Feb 18;64:102050. doi: 10.1016/j.gore.2026.102050 (PMC12938156; doi:10.1016/j.gore.2026.102050)

**Supplemental Figure 1A-F A Monte Carlo sensitivity analysis was performed to predict the reliability of each model according to a willingness-to-pay threshold determined by the ICER.**

Supplemental Figure 1AI. (PEMTC vs. TC, dMMR)


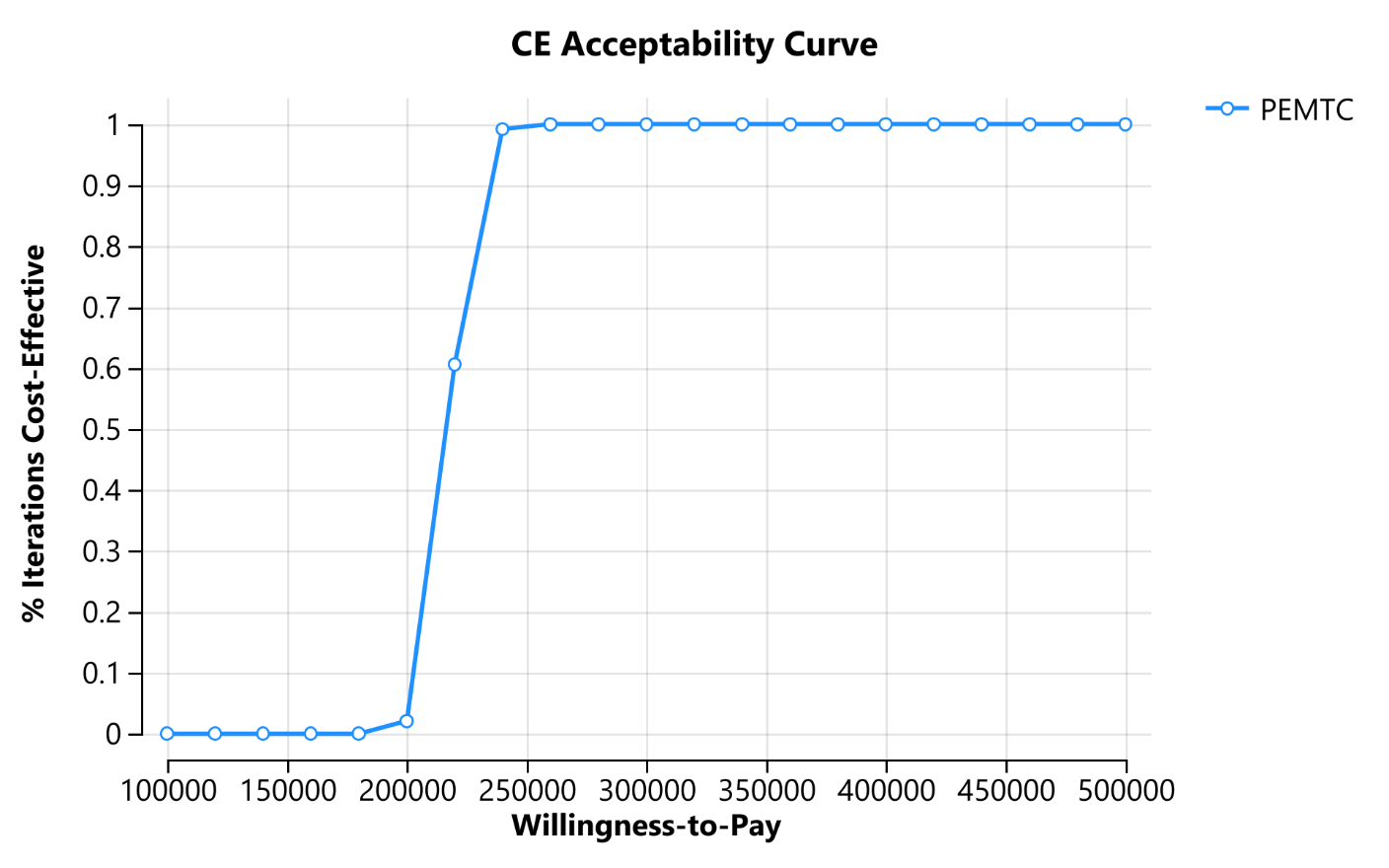


Supplemental Figure 1AII. (PEMTC vs. TC, dMMR)


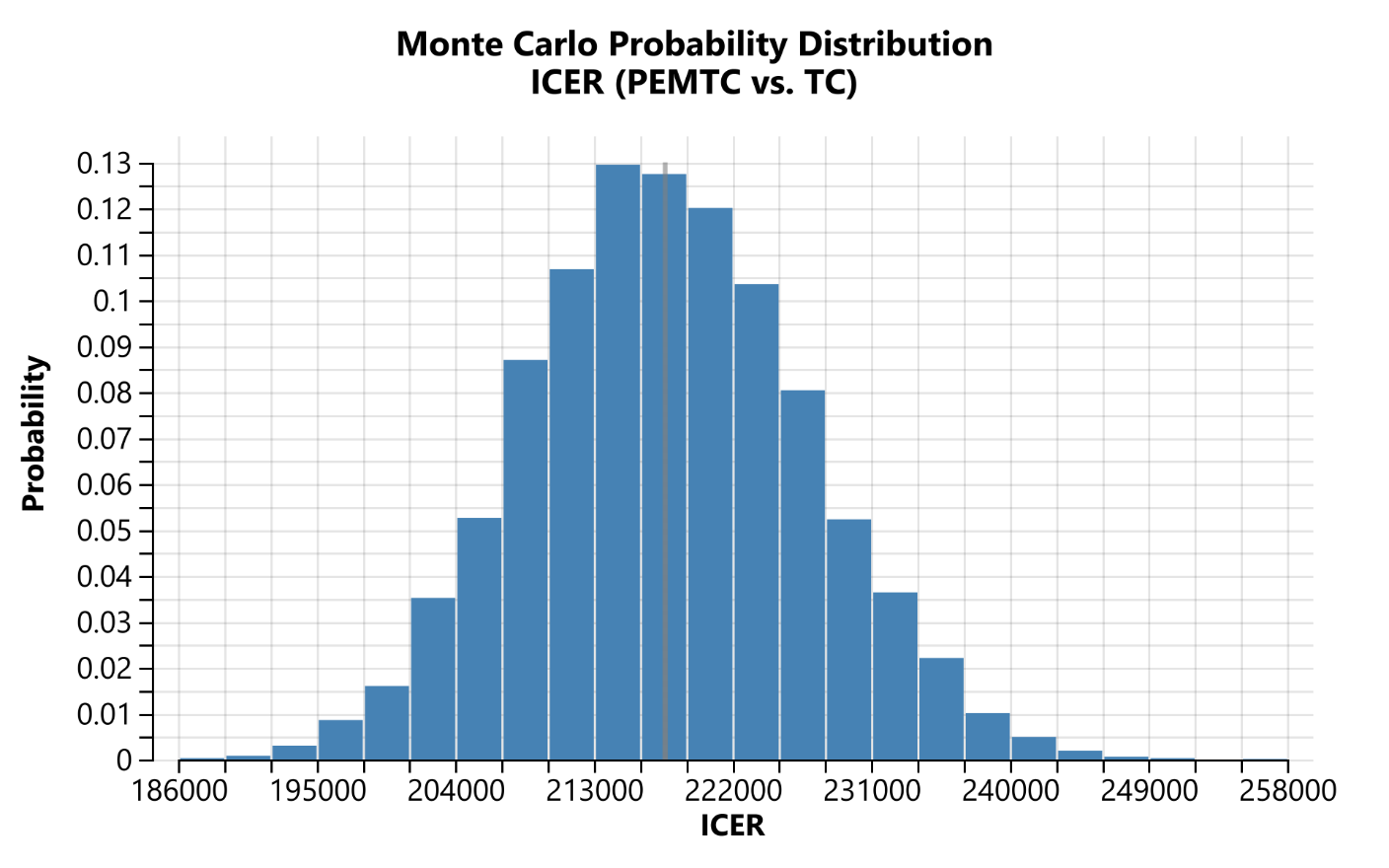


Supplemental Figure 1BI. (PEMTC vs. TC, pMMR)


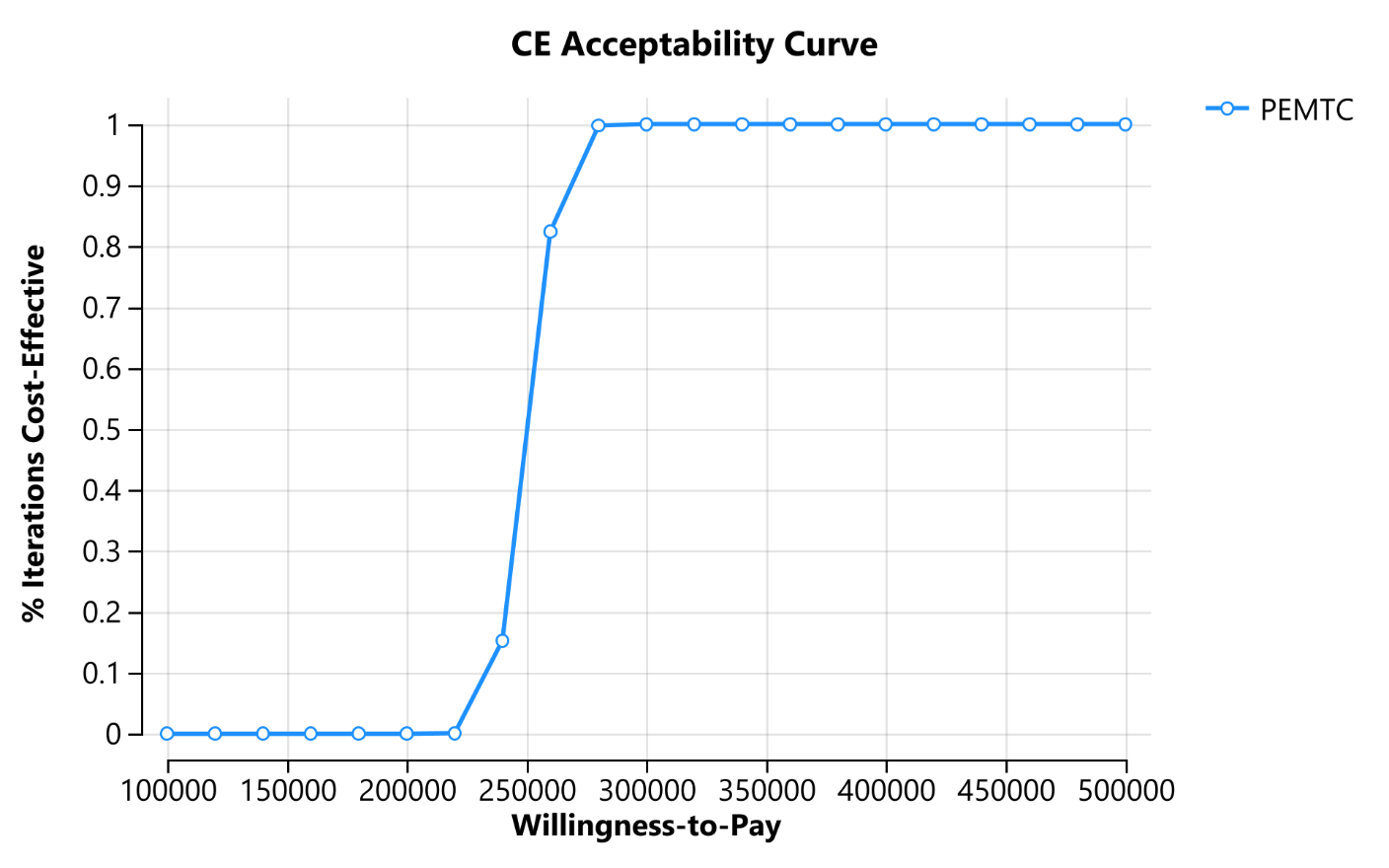


Supplemental Figure 1BII. (PEMTC vs. TC, pMMR)


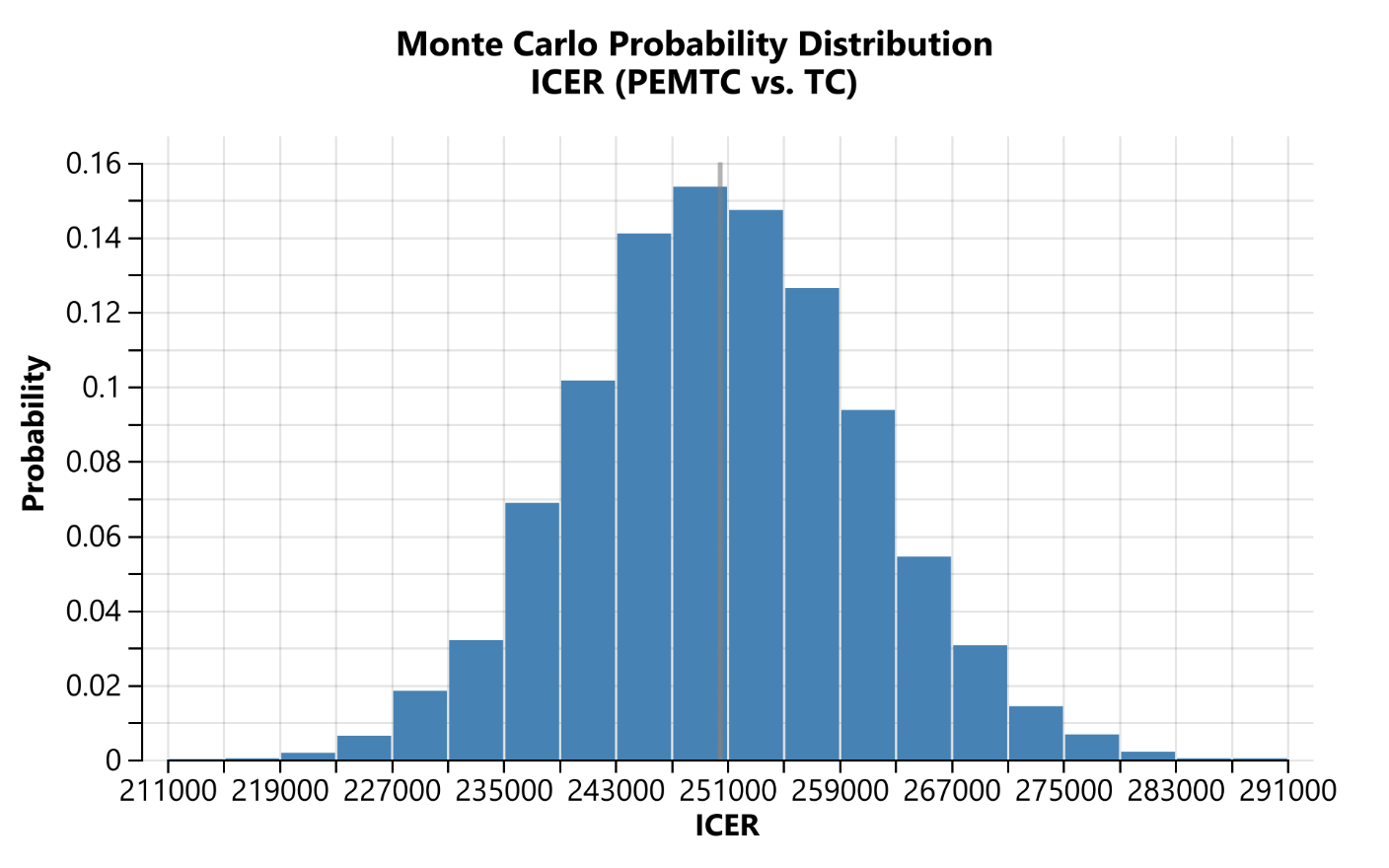


Supplemental Figure 1CI. (DOSTC vs. TC, dMMR)


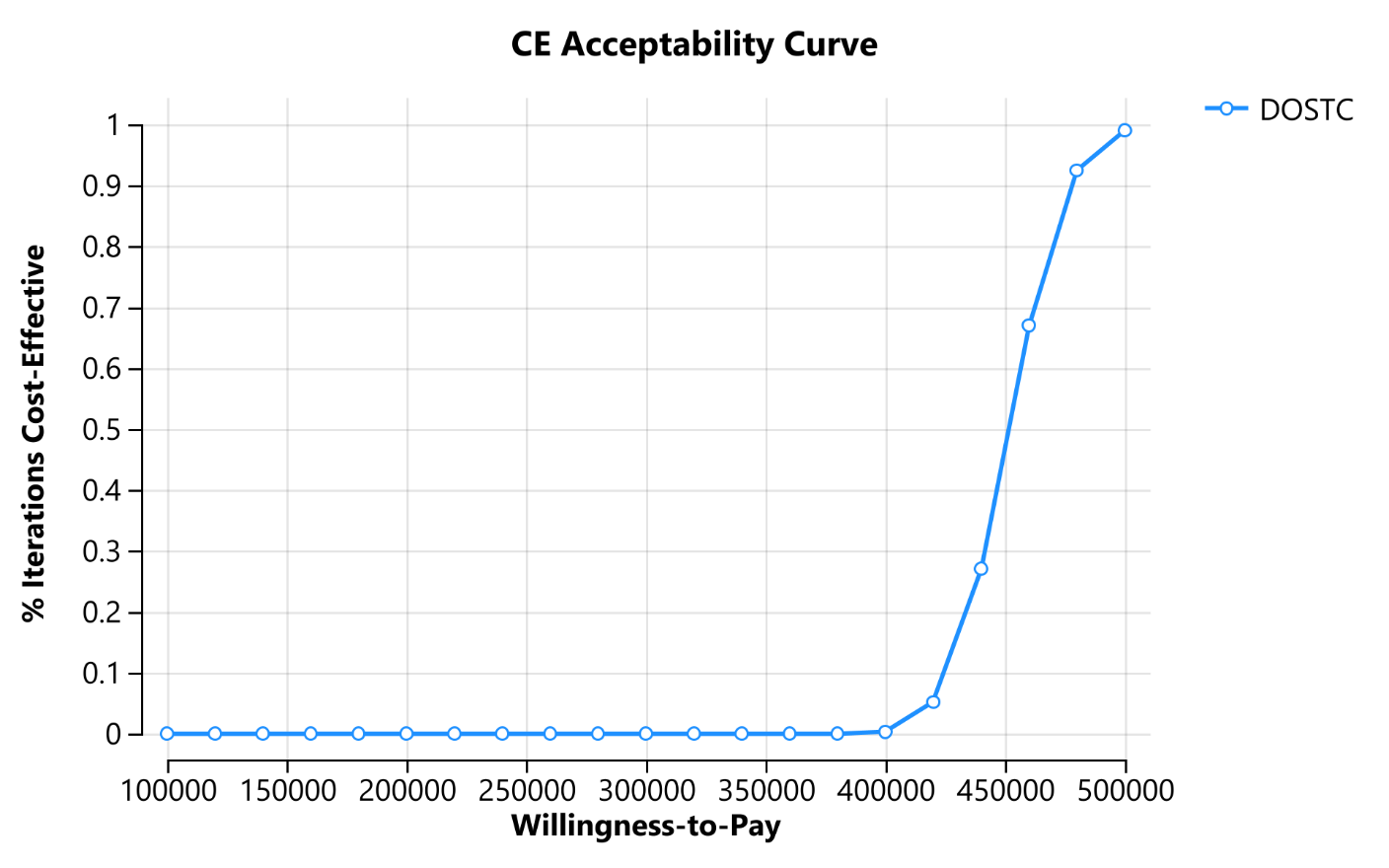


Supplemental Figure 1CII. (DOSTC vs TC, dMMR)


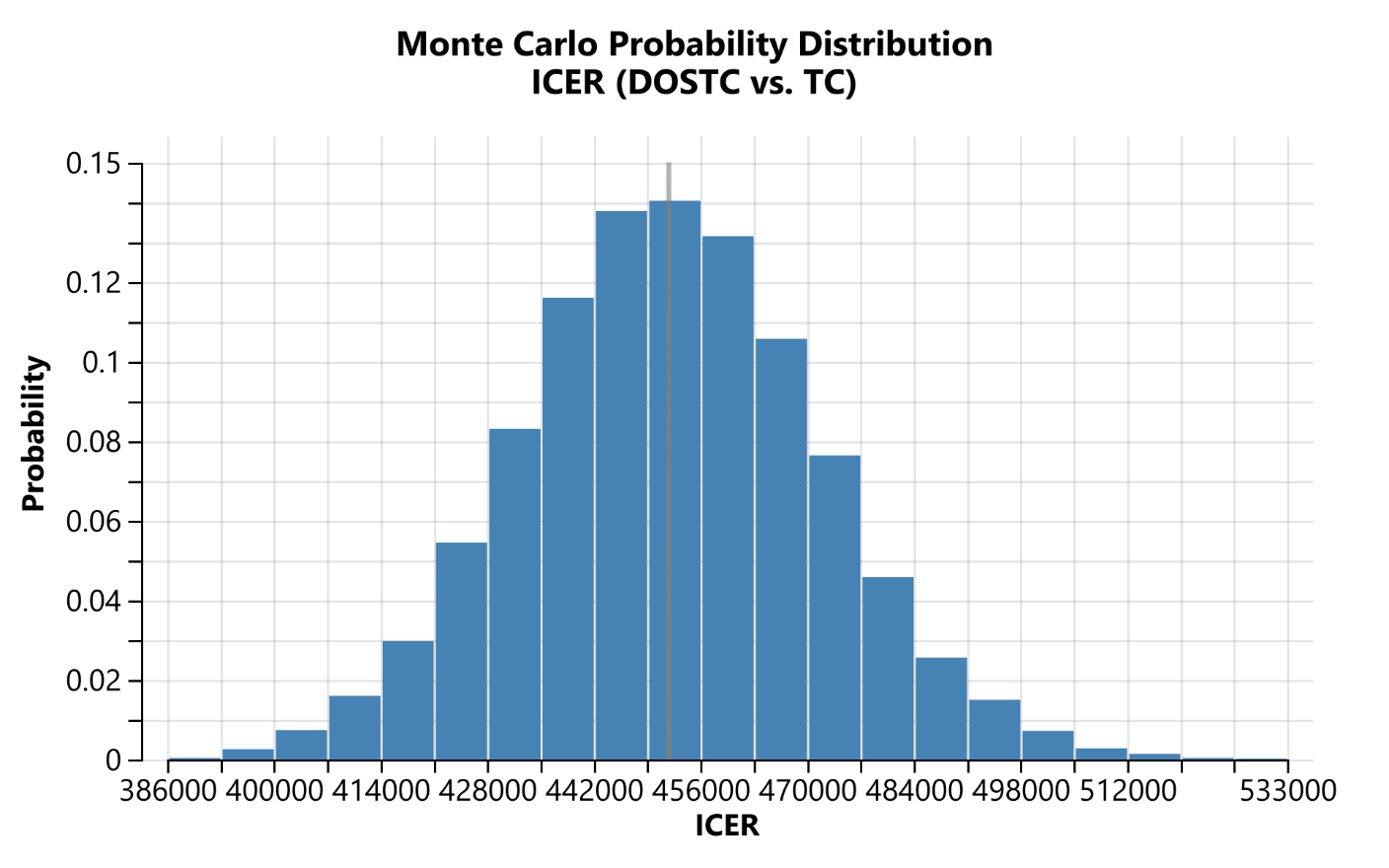


Supplemental Figure 1DI. (DOSTC vs. TC, pMMR)


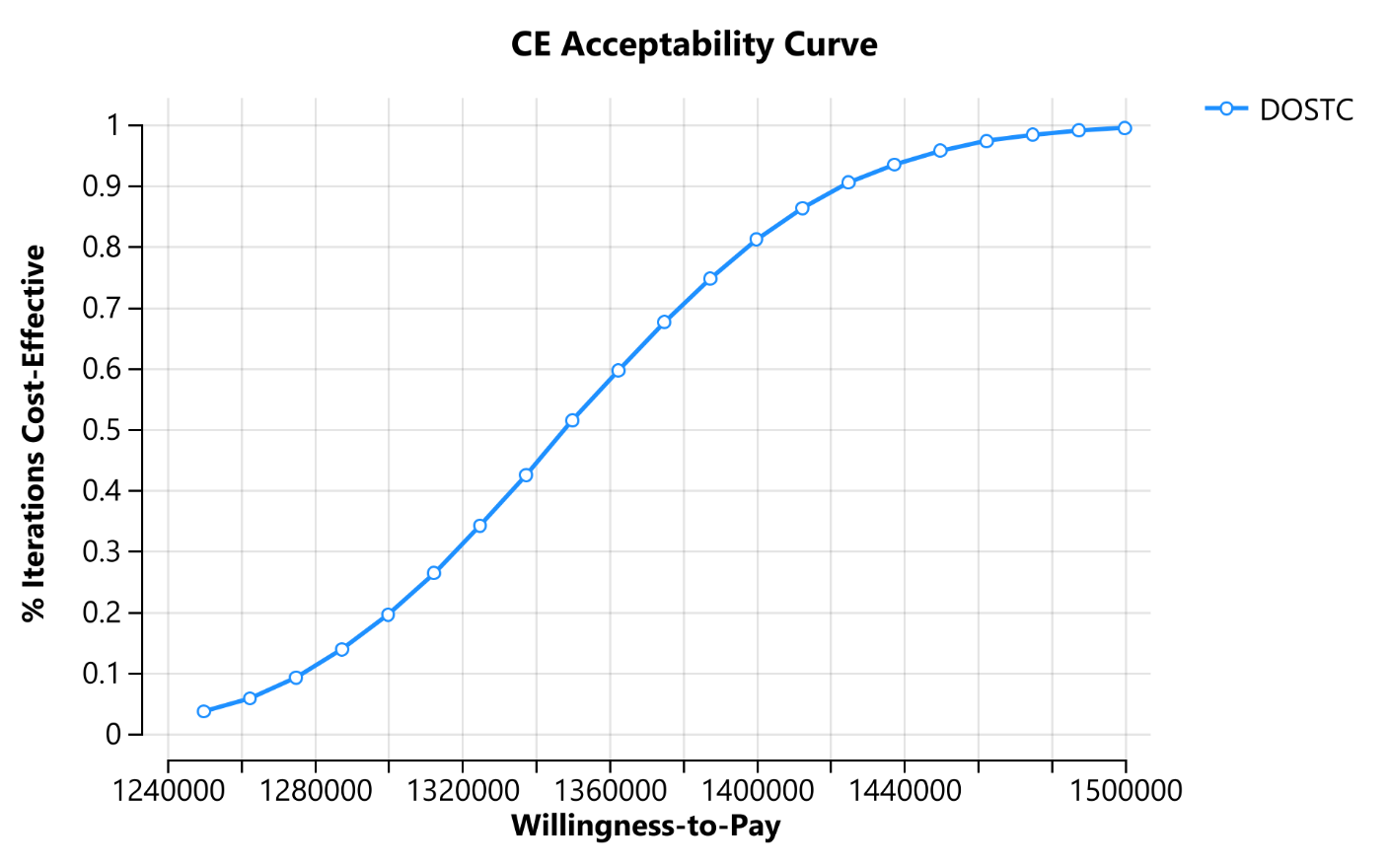


Supplemental Figure 1DII. (DOSTC vs. TC, pMMR)


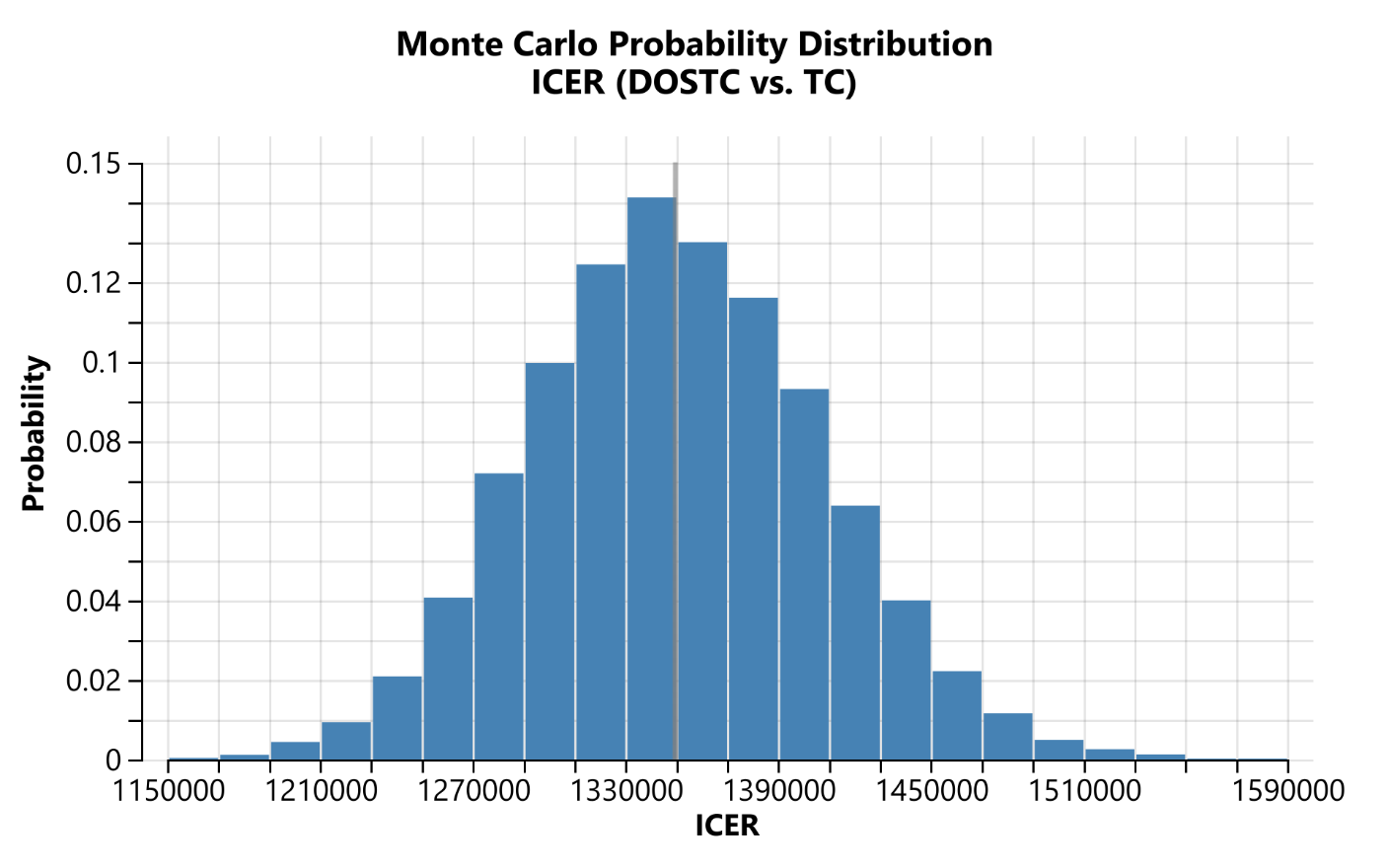


Supplemental Figure 1EI. (DURTC vs. TC, 2-year)


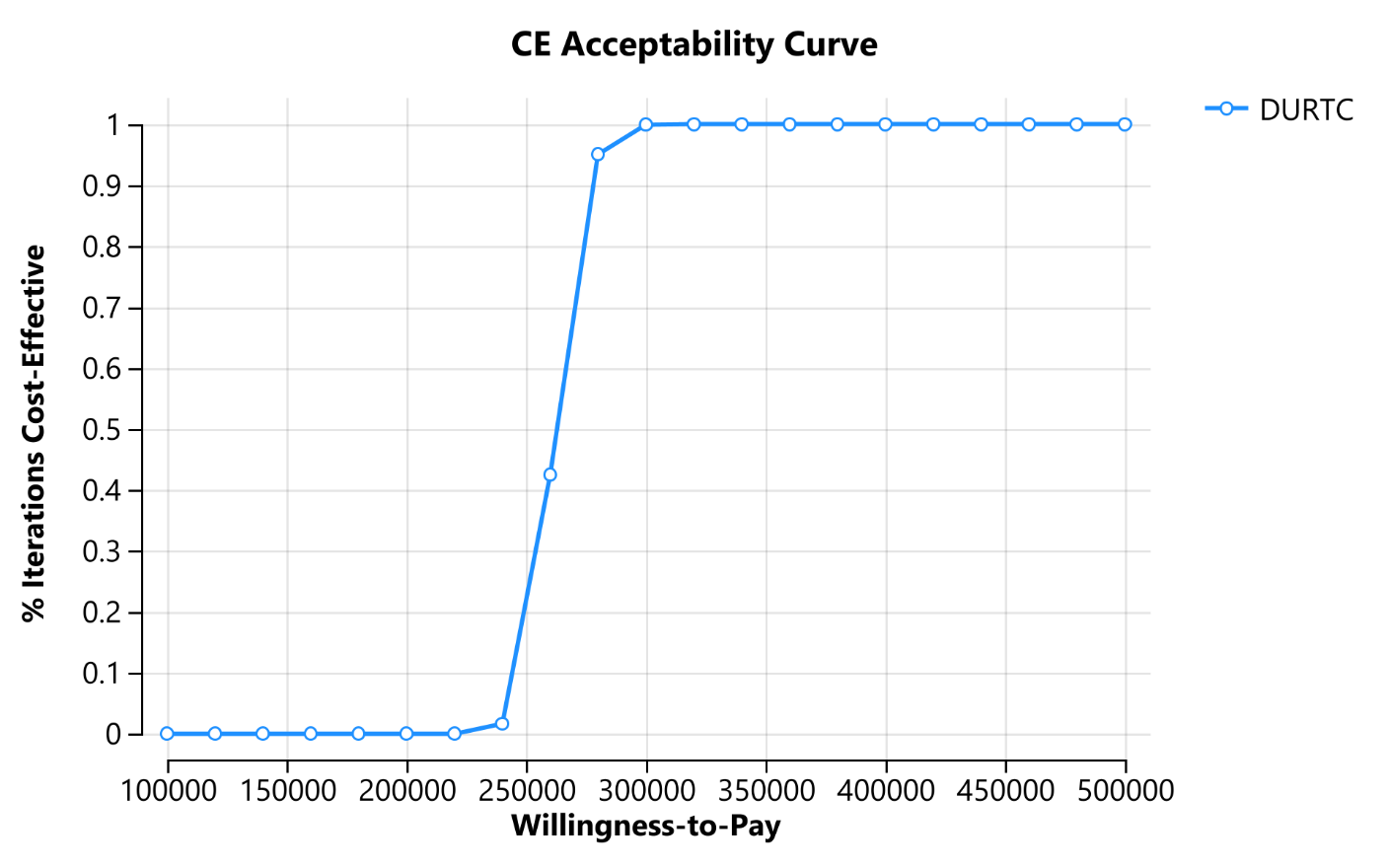


Supplemental Figure 1EII. (DURTC vs. TC, 2-year)


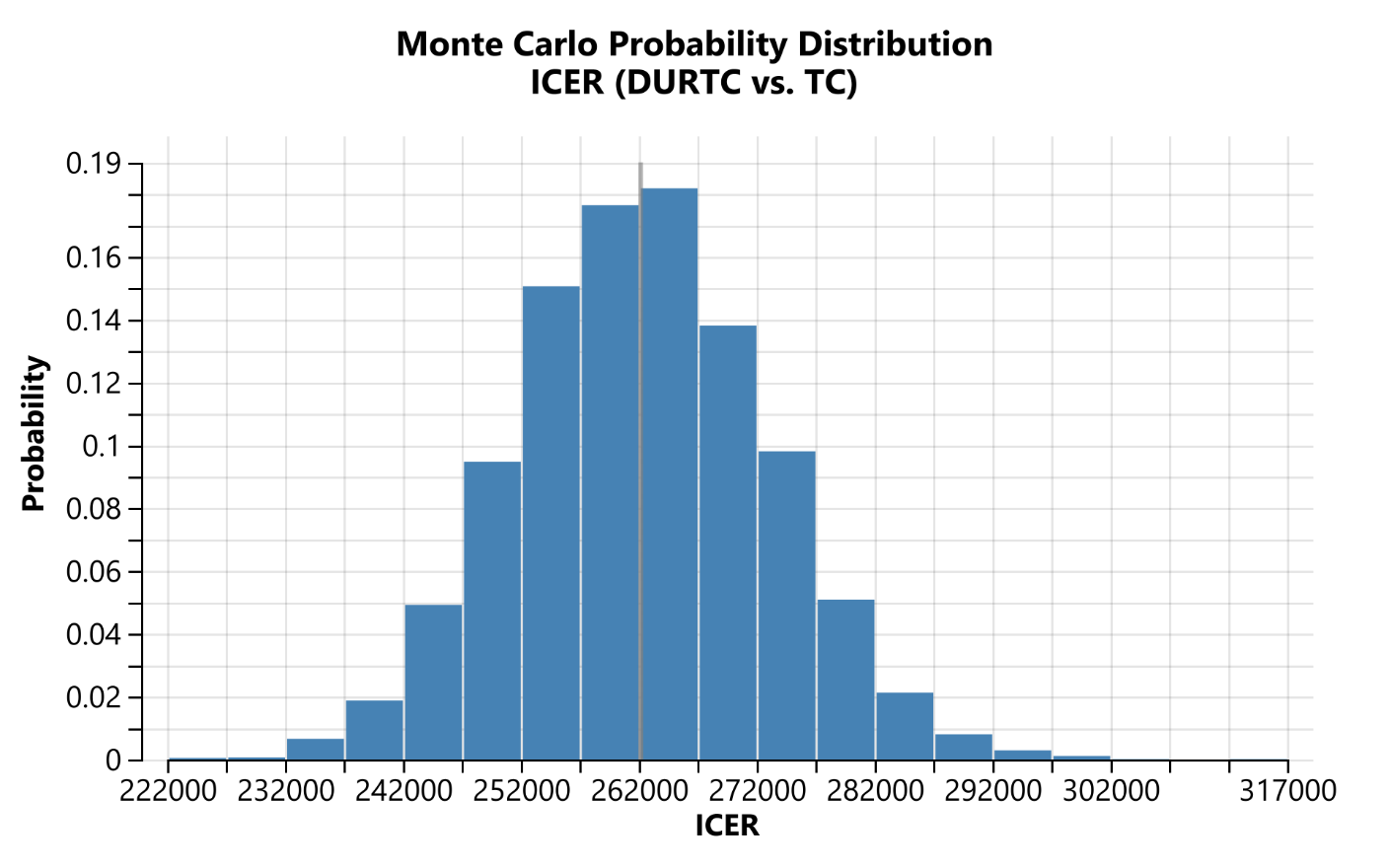


Supplemental Figure 1FI. (DURTC vs. TC, 3-year)


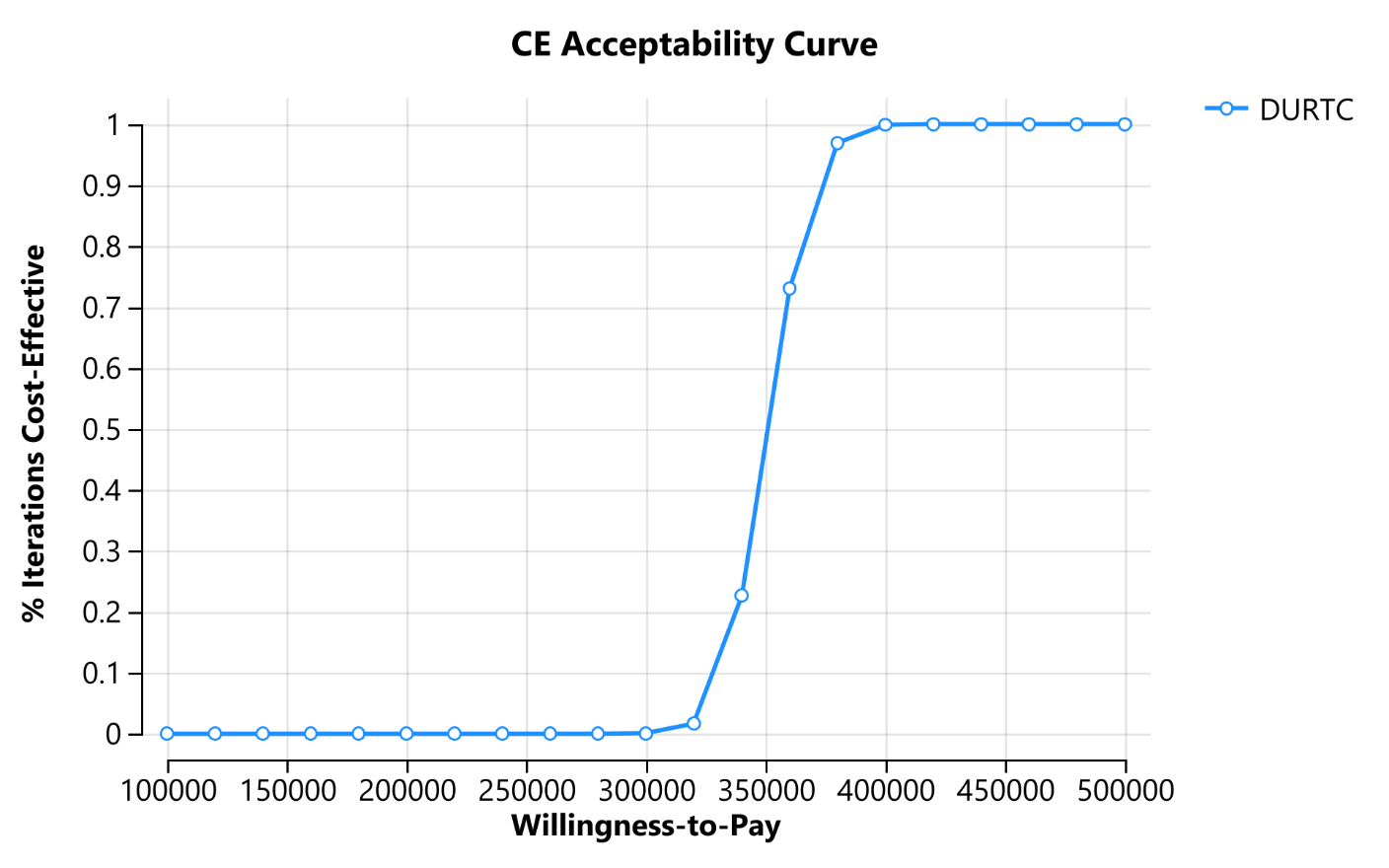


Supplemental Figure 1FII. (DURTC vs. TC, 3-year)


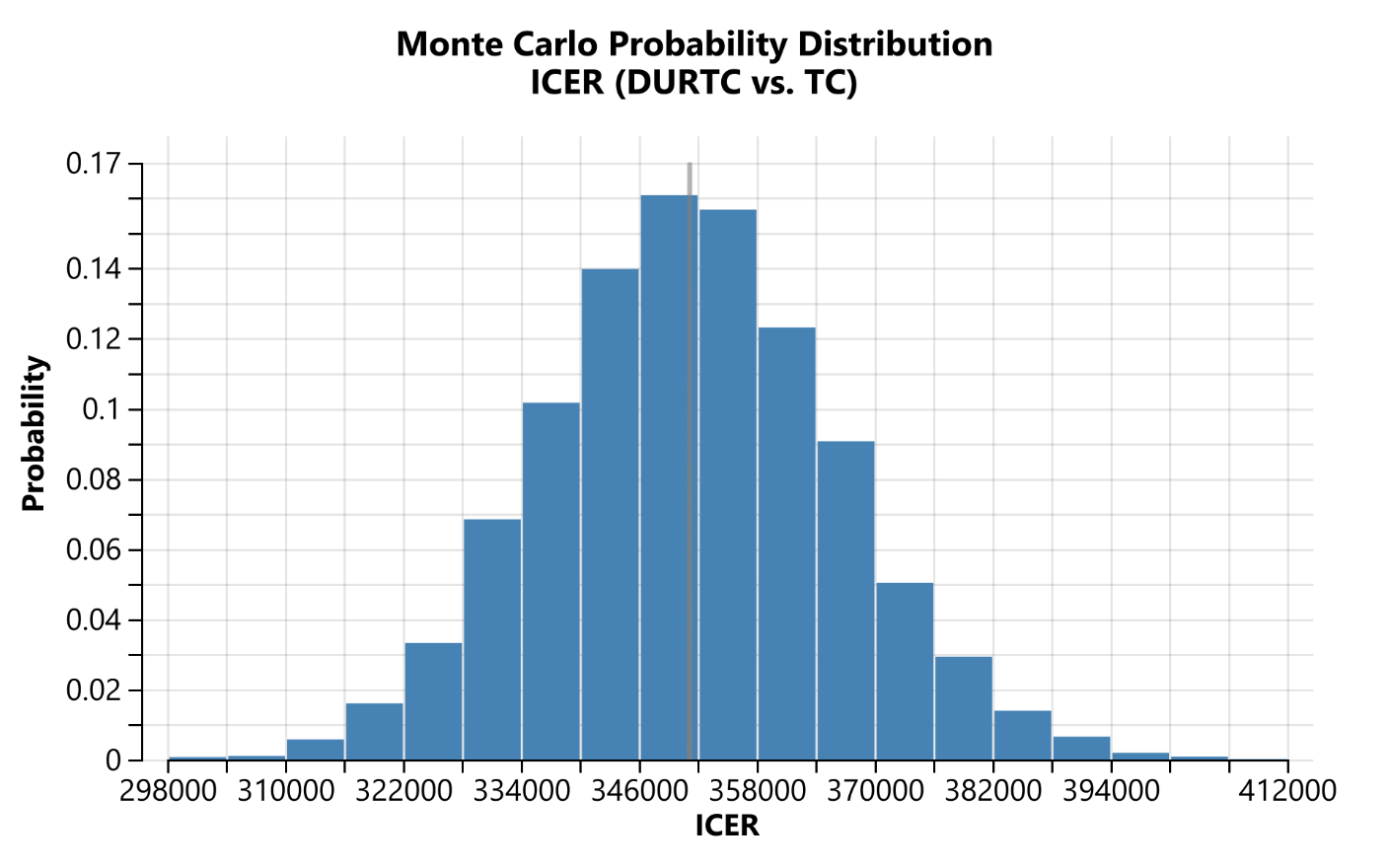


**Figure 4A-F. A tornado diagram displaying the results of the univariate sensitivity analysis for each model with inputs including costs of regimens, response rates, costs for toxicities, and health utility values.**

Supplemental Figure 2A. Tornado diagram for dMMR PEMTC vs. TC


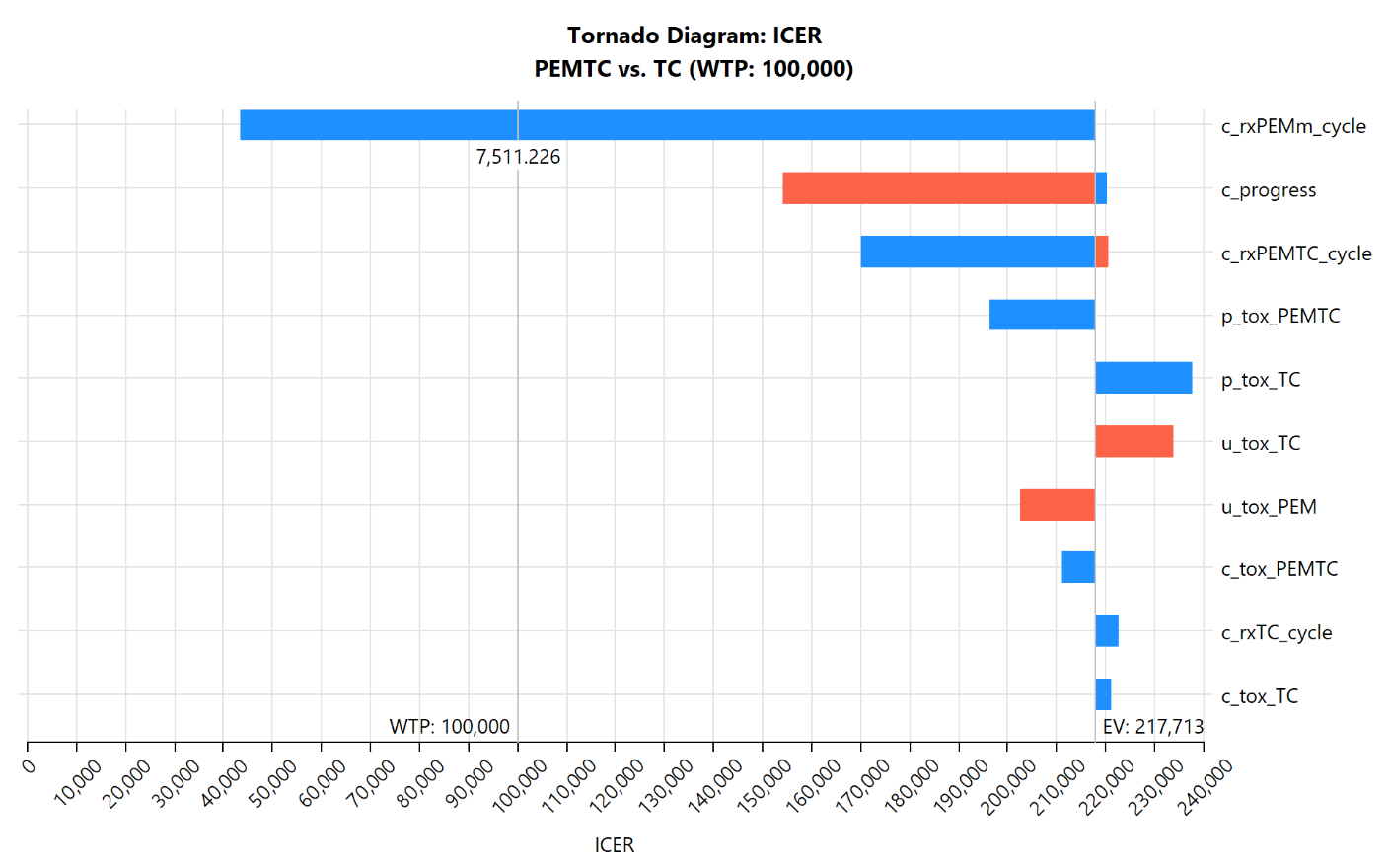


Supplemental Figure 2B. Tornado diagram for pMMR PEMTC vs. TC


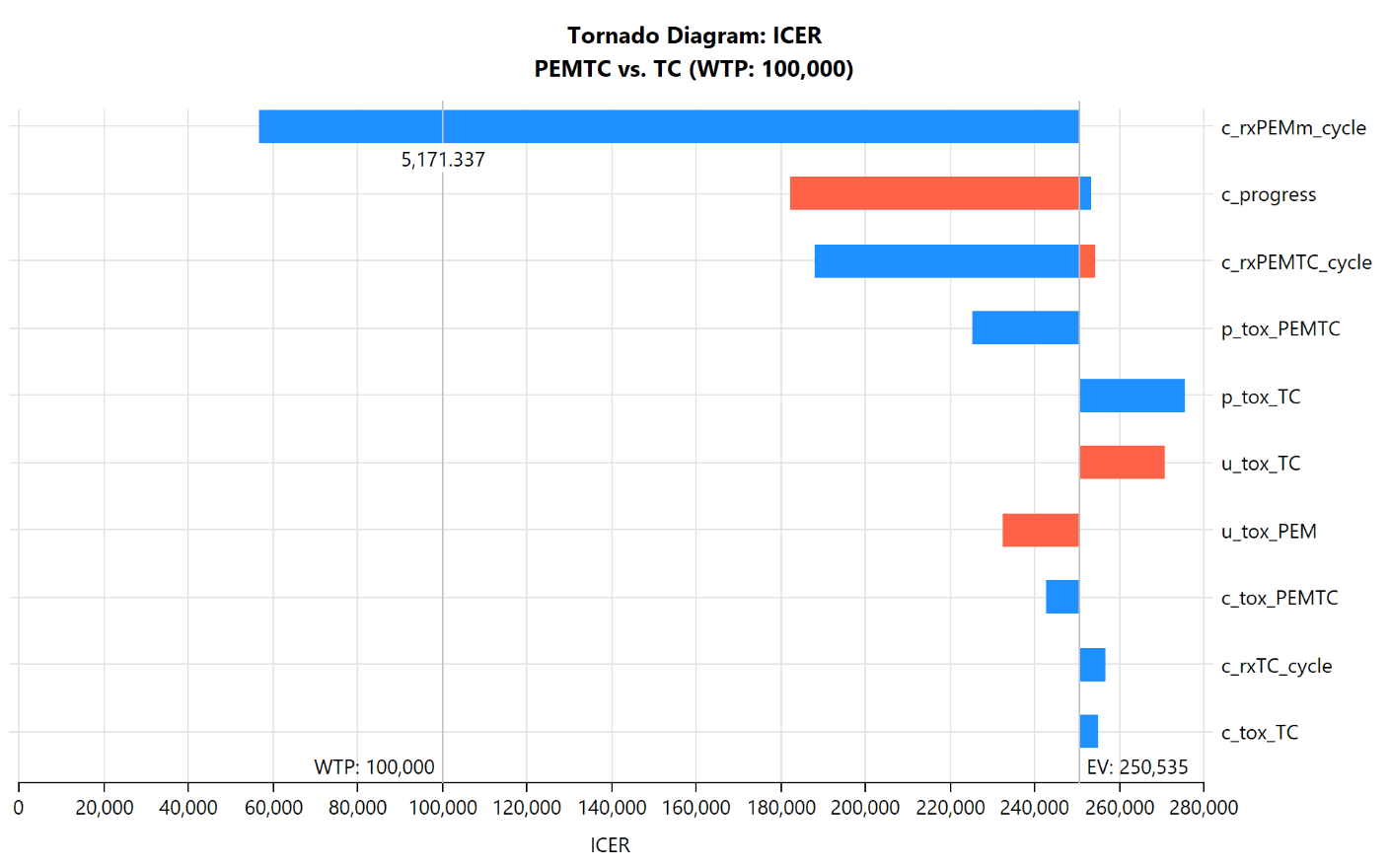


Supplemental Figure 2C. Tornado diagram for dMMR DOSTC vs. TC


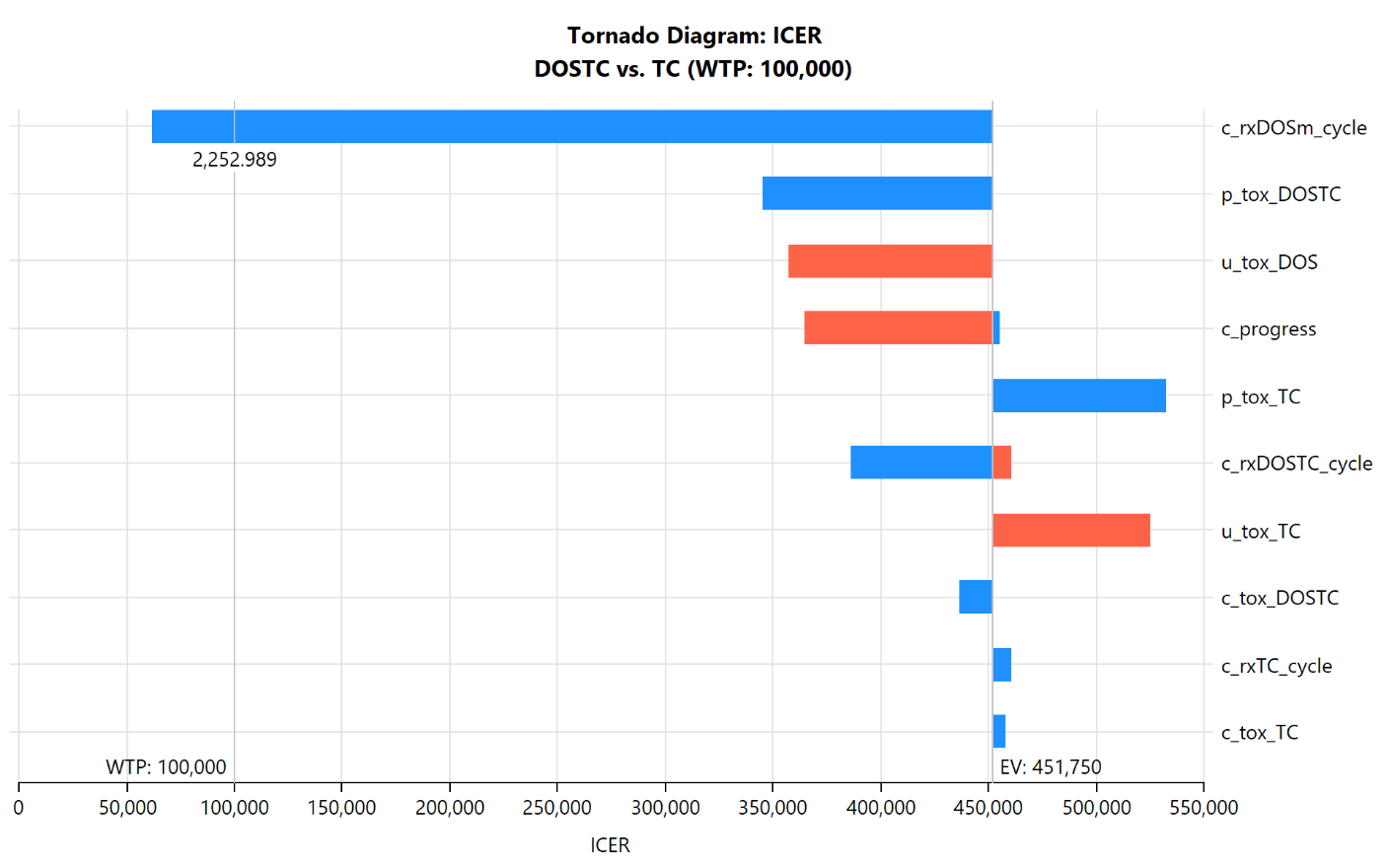


Supplemental Figure 2D. Tornado diagram for pMMR DOSTC vs. TC


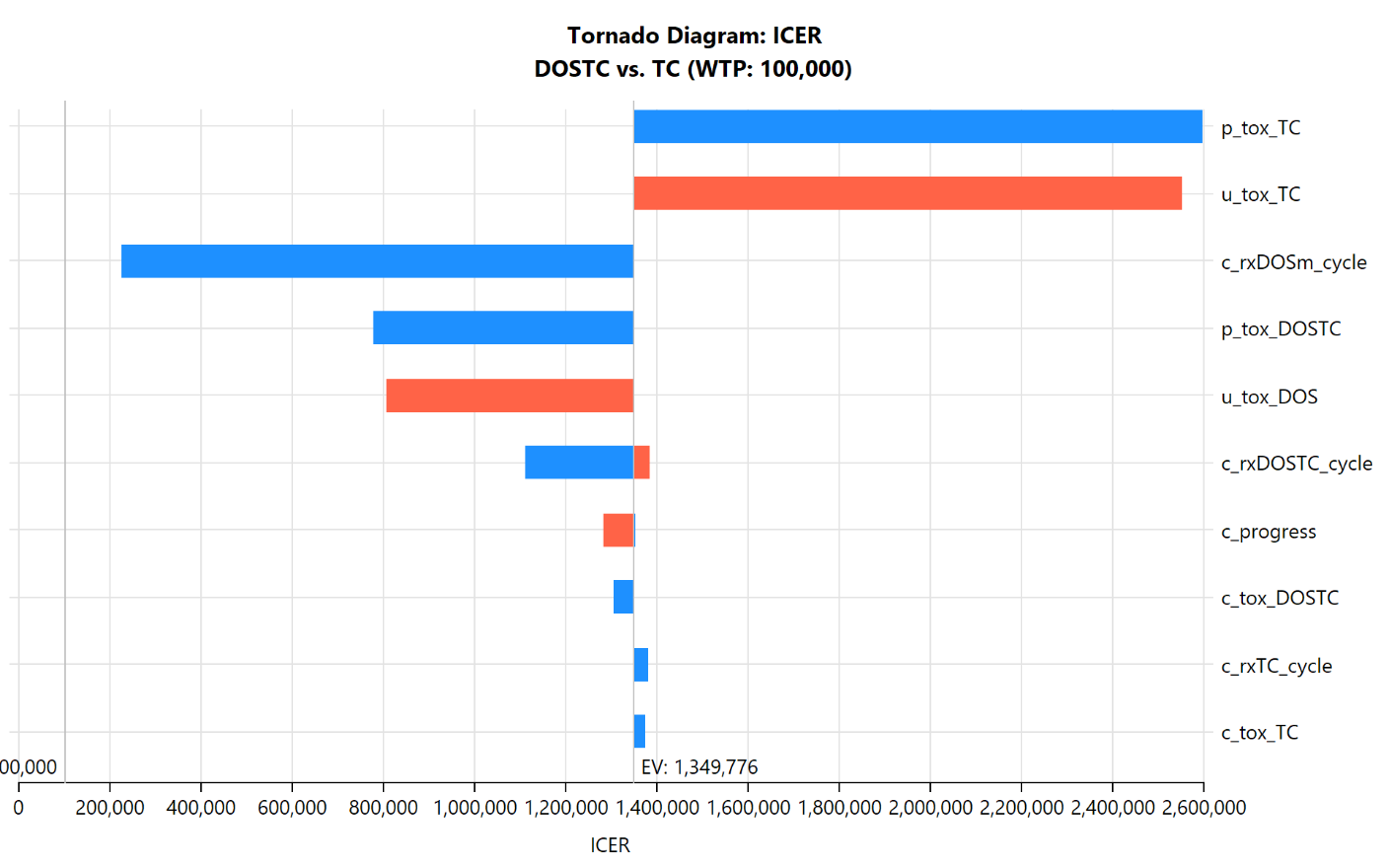


Supplemental Figure 2E. Tornado diagram for dMMR 3 year DURTC vs. TC


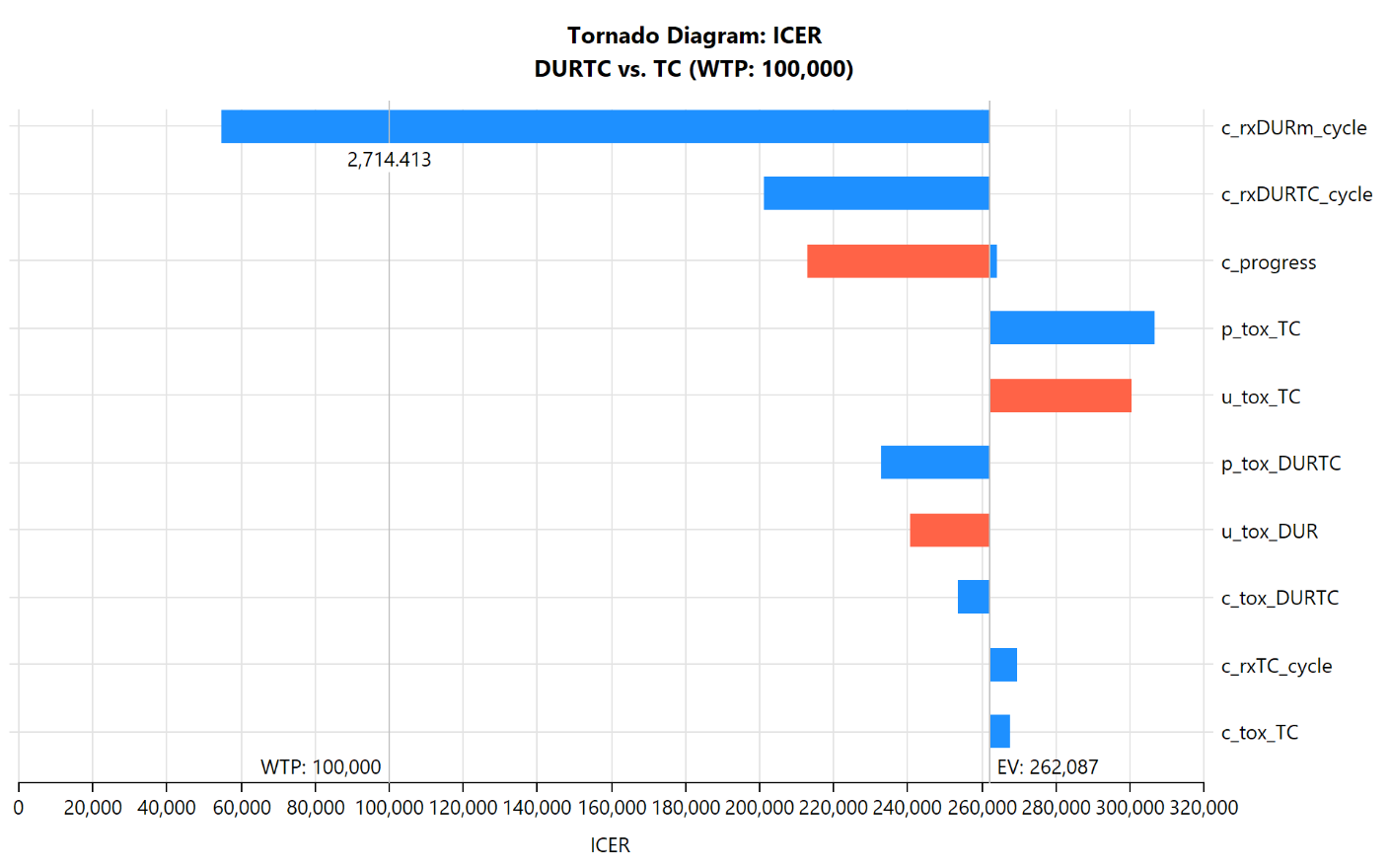


Supplemental Figure 2F. Tornado diagram for dMMR 2 year DURTC vs. TC


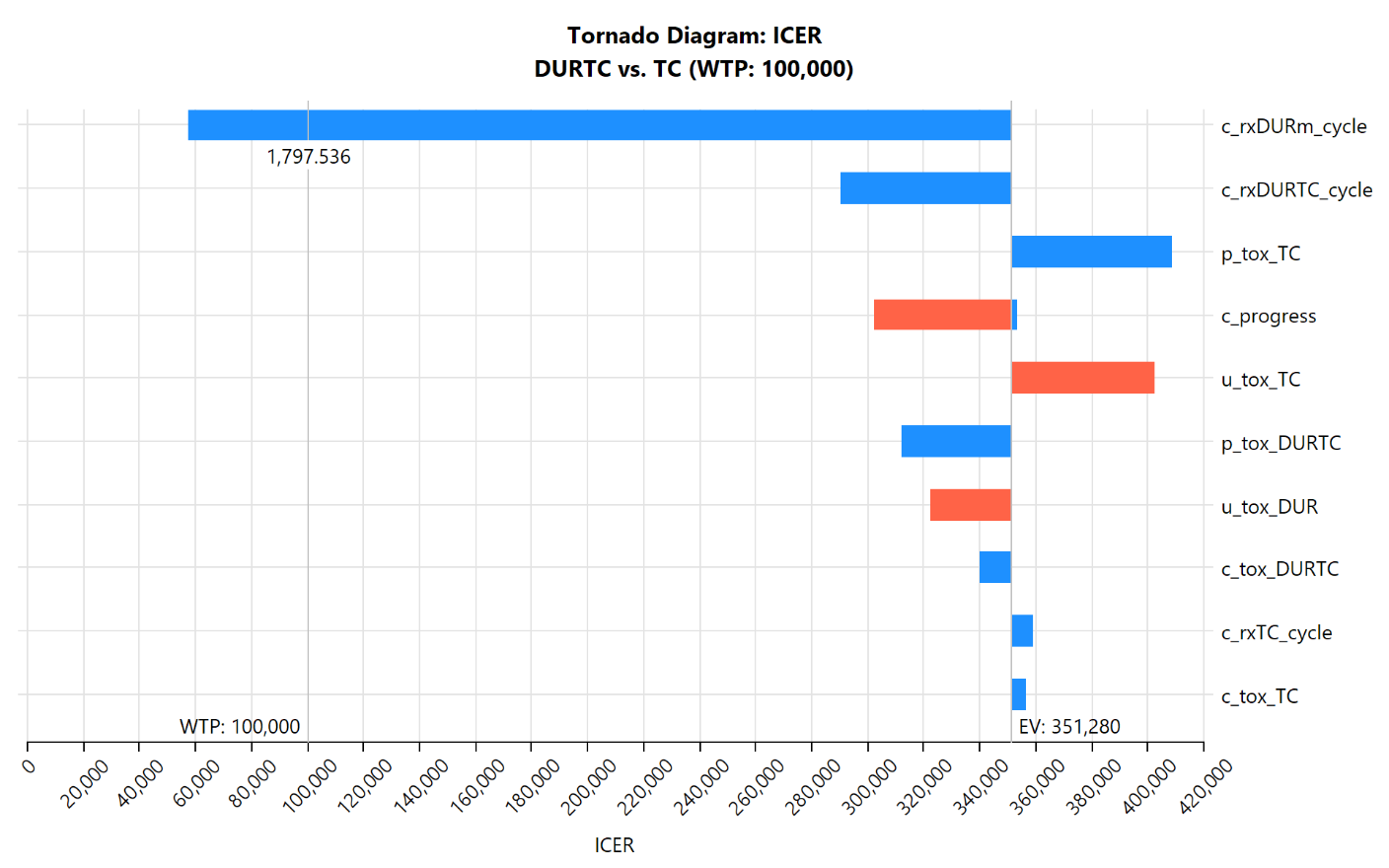

Supplement: Supplementary Data 1 [file mmc1.docx]
